# Mass Citationism: A Deleterious Measure of Scientific Value

**DOI:** 10.3390/tomography12090119

**Published:** 2026-08-24

**Authors:** Emilio Quaia

**Affiliations:** Department of Radiology, University of Padova, Via Giustiniani 2, 35128 Padova, Italy; emilio.quaia@unipd.it; Tel.: +39-049-8212375

This editorial examines the phenomenon of mass citationism, often critiqued as using bibliometrics as “weapons of mass citation” [1,2] and defined as the over-reliance on citation counts and quantitative indices (such as h-index) to evaluate the quality of scientific research, academic careers, and institutional funding, often treating these metrics as definitive evidence rather than as tools that complement critical analysis, original argumentation, and genuine scholarly debate.

Science advances through the generation of new knowledge, rigorous debate, and the continuous verification of evidence. Citations have traditionally served as the intellectual threads connecting discoveries across generations, acknowledging prior contributions while allowing scientific ideas to evolve. Yet, in recent decades, citations have progressively acquired a function far beyond scholarly attribution. They have become a currency of academic success. The consequence is the emergence of what may be called mass citationism, namely the systematic overreliance on citation counts as the principal measure of scientific value. The allocation of resources for research is increasingly based on so-called ‘bibliometrics’. Scientists are now considered successful only if their work is widely cited [2]. This worldwide trend appears to enjoy support not only from granting agencies, but also by the scientists themselves who seem to enjoy their status of celebrities [2].

Mass citationism should not be confused with the legitimate practice of citing previous work. Rather, it represents a cultural and institutional transformation in which bibliometric indicators increasingly substitute for critical judgment. Citationism has profoundly reshaped academic evaluation systems, transforming citations from simple tools of scientific attribution into genuine indicators of professional value. The scientific value of a researcher, a journal, or even an entire institution is often reduced to numerical indices such as the h-index, Impact Factor, CiteScore, or institutional citation rankings. While these metrics were originally introduced as descriptive tools, they have gradually evolved into normative standards that shape careers, funding decisions, promotions, and research priorities.

This transformation has profound consequences. Scientific quality is a multidimensional concept encompassing originality, methodological rigor, reproducibility, societal relevance, and long-term impact. Citation counts capture only a narrow aspect of this complexity. Moreover, citations are not synonymous with scientific excellence. A paper may receive hundreds of citations because it introduces a revolutionary concept, but it may also be cited because it contains controversial claims, methodological flaws, or even fraudulent results. Current bibliometric systems rarely distinguish between positive recognition and critical citation. Consequently, criticism and endorsement often generate identical bibliometric rewards. This phenomenon shifts the focus from the intrinsic quality of research to the quantity of citations received, thereby distorting the entire ecosystem of scientific evaluation.

*First*, this trend distorts the core metrics used for research assessment, particularly the h-index and the Impact Factor. Current research assessment systems rely on quantitative indicators such as the h-index (for individual researchers) and the Impact Factor (for scientific journals), which are calculated and reported by databases such as Clarivate’s Web of Science and Elsevier’s Scopus. This system effectively equates criticism with endorsement, since these algorithms count every citation equally. An article cited negatively because it contains errors receives the same citation credit as an article praised for a groundbreaking discovery. Moreover, citationism incentivizes quantity over quality, as researchers are encouraged to divide their work into multiple shorter papers—a practice known as salami slicing—to increase the number of opportunities for their work to be cited.

*Second*, there has been a clear emergence of strategic and manipulative practices. In the effort to climb national research evaluation rankings and university league tables, a range of artificial behaviors has emerged with the aim of inflating citation metrics. These include citation cartels (or citation rings), in which groups of researchers systematically agree to cite one another’s work regardless of its actual scientific relevance; excessive self-citation, whereby authors repeatedly cite their own previous publications even when the thematic connection is weak or nonexistent; and coercive citation, in which journal editors or peer reviewers pressure authors to add citations to articles published in the same journal as a condition for acceptance, thereby artificially increasing the journal’s Impact Factor.

The growing dependence on citation metrics has also reshaped researchers’ behavior. Rather than pursuing ambitious, long-term scientific questions, investigators may increasingly select topics likely to generate rapid visibility and high citation counts. These incentives encourage strategic publishing practices and reinforce the conversion of citations into academic capital. The consequences extend beyond individual researchers. Universities increasingly compete in international rankings that heavily incorporate citation-based indicators. Funding agencies frequently rely on bibliometric evidence to evaluate grant applications, while promotion committees often consider citation metrics as objective measures of scientific productivity. The result is a self-reinforcing system in which quantitative performance becomes an end in itself. As Goodhart’s Law states, *when a measure becomes a target, it ceases to be a good measure*. Citation counts illustrate this principle with remarkable clarity.

Another concern is the unequal distribution of citation opportunities across scientific disciplines. Biomedical sciences and artificial intelligence naturally generate far larger citation networks than mathematics, philosophy, or many areas of the humanities. Similarly, English-language publications, large international collaborations, and well-established institutions enjoy structural advantages in citation accumulation. Consequently, citation metrics frequently measure visibility and disciplinary dynamics rather than intrinsic scientific merit. Mass citationism also influences the dissemination of scientific knowledge. Researchers may preferentially cite highly visible papers instead of the most rigorous or original ones whereby already well-known authors accumulate even greater recognition simply because they have already achieved recognition. This positive feedback mechanism concentrates scientific prestige and limits the diversity of perspectives entering academic debate.

The solution is not to abandon bibliometric indicators altogether. Citations remain valuable instruments for mapping scientific influence and intellectual networks. However, they should be interpreted within a broader framework of qualitative evaluation. Peer review, methodological transparency, reproducibility, societal impact, open science practices, and genuine scientific innovation must regain a central role in research assessment.

Ultimately, science should reward ideas rather than metrics. A research system that equates scientific excellence with citation accumulation risks encouraging strategic publishing behavior instead of intellectual creativity. If citations become the primary objective rather than the natural consequence of meaningful discoveries, the scientific enterprise risks confusing popularity with quality.

Mass citationism is therefore not merely a bibliometric phenomenon; it is a cultural transformation affecting the values upon which modern science is built. Restoring scientific integrity requires recognizing that no numerical indicator, however sophisticated, can fully capture the richness, originality, and societal importance of scientific knowledge. Citations should remain what they were always intended to be: evidence of scholarly dialog rather than the ultimate measure of scientific value.

International initiatives such as the San Francisco Declaration on Research Assessment (DORA) [3] and the Leiden Manifesto [4] have called for a more responsible use of bibliometric indicators, emphasizing that metrics should support—not replace—expert judgment. Reversing the current trend requires a renewed commitment to the principles of rigorous peer review and to the values of genuine scientific inquiry. Bibliometric indicators should inform evaluation rather than dictate it, restoring human expertise and critical judgment to the center of research assessment.
